# Avian ANP32A incorporated in avian influenza A virions promotes interspecies transmission by priming early viral replication in mammals

**DOI:** 10.1126/sciadv.adj4163

**Published:** 2024-01-31

**Authors:** Lei Na, Liuke Sun, Mengmeng Yu, Yingzhi Zhang, Yuan Zhang, Zhenyu Zhang, Haili Zhang, Ting Qi, Wei Guo, Xing Guo, Shida Wang, Jingfei Wang, Yuezhi Lin, Xiaojun Wang

**Affiliations:** ^1^ State Key Laboratory for Animal Disease Control and Prevention, Harbin Veterinary Research Institute, The Chinese Academy of Agricultural Sciences, Harbin 150069, China.; ^2^ Key Laboratory of Zoonosis Research, Ministry of Education, College of Veterinary Medicine, Jilin University, Changchun 130062, China.; ^3^ Institute of Western Agriculture, The Chinese Academy of Agricultural Sciences, Harbin 150069, China.

## Abstract

Species-specific differences in acidic nuclear phosphoprotein 32 family member A (ANP32A) determine the restriction of avian-signature polymerase in mammalian cells. Mutations that evade this restriction, such as PB2-E627K, are frequently acquired when avian influenza A viruses jump from avian hosts to mammalian hosts. However, the mechanism underlying this adaptation process is still unclear. Here, we report that host factor ANP32 proteins can be incorporated into influenza viral particles through combination with the viral RNA polymerase (vPol) and then transferred into targeted cells where they support virus replication. The packaging of the ANP32 proteins into influenza viruses is dependent on their affinity with the vPol. Avian ANP32A (avANP32A) delivered by avian influenza A virions primes early viral replication in mammalian cells, thereby favoring the downstream interspecies transmission event by increasing the total amount of virus carrying adaptive mutations. Our study clarifies one role of avANP32A where it is used by avian influenza virus to help counteract the restriction barrier in mammals.

## Teaser

Avian influenza A viruses incorporate avian ANP32A into their virions to help them adapt to mammalian hosts.

## INTRODUCTION

Influenza A viruses (IAVs) infect a broad range of avian and mammalian species. Wild water birds are considered to be a natural reservoir for avian IAVs (AIVs) (*1*). Mammalian hosts show restriction to AIVs. AIVs such as H5N1 and H7N9 circulate in different avian species and can occasionally cross into mammals with evolutionary adaptive mutations on the viral genome (*2*–*10*). Amino acid changes in the hemagglutinin (HA) and polymerase of AIV, as well as nucleoprotein (NP), are the major viral determinants to overcome the mammalian cell barrier (*2*–*4*, *6*). The IAV viral RNA polymerase (vPol) is a heterotrimer composed of the polymerase acidic(PA), polymerase basic 2 (PB2), and polymerase basic 1 (PB1), which forms viral ribonucleoprotein (vRNP) complexes with NP and viral RNA (vRNA) and is responsible for the transcription and replication of viral genome (*11*–*13*). The activity of AIV vPol is restricted in human cells, and the species-specific differences in acidic nuclear phosphoprotein 32 family member A (ANP32A, pp32) between humans and birds were recently found to be the key factor accounting for this restriction (*14*–*16*).

ANP32 proteins are essential for influenza polymerase activity (*15*, *17*–*22*). Avian ANP32A specifically supports AIV vPol activity due to the 33–amino acid insert, but mammalian shorter ANP32A and ANP32B show only limited support to avian vPol activity (*15*, *18*). Subsequent studies suggest that there are three predominant isoforms of avANP32A that differ only in composition of the 33–amino acid insert. These three isoforms differ in their ability to support avian-signature vPol activity and have different effects on the adaptation of avian-signature vPol (*23*, *24*). Further research confirmed that the presence of a unique hydrophobic small ubiquitin-related modifier (SUMO) interaction motif-like sequence in the avian-specific 33–amino acid insertion determines the selectively supporting function of avANP32A toward avian-signature vPol activity, although the detailed mechanism is currently unknown (*25*). Avian ANP32B (avANP32B) is naturally inactive due to mutations at the key sites of 129 and 130 so that AIVs only use avANP32A to support their replication in avian hosts (*18*, *19*). In addition, other researchers and our laboratory have independently demonstrated that human ANP32A (huANP32A) and ANP32B (huANP32B) contribute equally to support the activity of human-adapted IAV vPol and that IAV is unable to replicate in the absence of both huANP32A and huANP32B (*18*, *20*). Owing to the unique 106V/156S signature, swine ANP32A supports the avian-signature vPol activity to a greater extent as compared with other mammalian shorter ANP32A and ANP32B, providing a molecular basis of interspecies transmission of AIVs between chickens and pigs (*26*, *27*). In addition, our recent study provides evidence that ANP32 proteins are also decisive host factors for influenza B virus (IBV) polymerase activity (*28*).

Studies have shown ANP32 proteins support IAV vPol activity by associating with vPol (*18*–*20*, *25*, *29*). ANP32 proteins can mediate two polymerase complex molecules to form an asymmetric dimer, thus providing a replication platform (*30*). To date, the specific molecular mechanism that triggers the replication and adaptation of AIV, especially how the AIV can initiate the early stage of replication under the restriction of mammalian ANP32A/B when they first infect mammalian cells, is largely unknown. Given that ANP32 proteins have a strong interaction with the vPol and that only very small amounts of ANP32 are required for efficient replication of the IAV (*18*), we hypothesized that avANP32A proteins could be packaged into AIV, where it promotes initial viral replication, and further benefits viral adaptation when AIVs jump from avian species into mammals. In this report, we confirm that host factor ANP32 proteins are incorporated into both IAV and IBV, and are transferred into newly infected cells upon virus infection to support viral replication. The packaging of ANP32 proteins by the influenza virus depends on the affinity between vPol and ANP32 proteins, regardless of its ability to support viral vPol activity. We found that incorporation of avANP32A, but not huANP32A, could prime the early replication of AIV when infecting mammalian cells both in vivo and in vitro. Moreover, the enhanced replication conferred by the transferred avANP32A may benefit the acquisition of mammalian-adaptive mutations in the viral genome. These findings reveal a crucial role for avANP32A in promoting the interspecies transmission and host adaptation of AIV.

## RESULTS

### ANP32 proteins are incorporated into influenza A viral particles

**Fig. 1.**
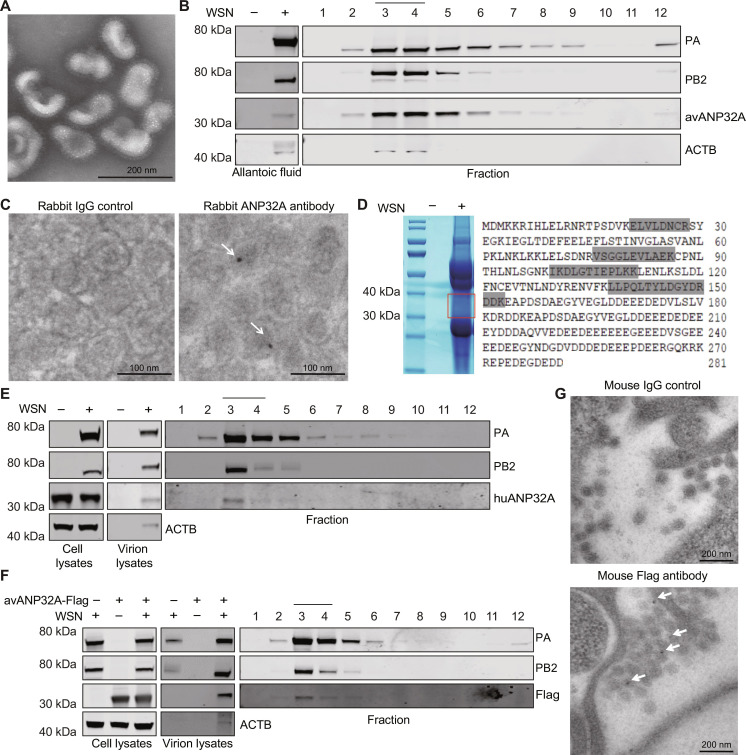
ANP32 proteins are packaged in influenza A viral particles. (**A**) Purified WSN virions produced from embryonated chicken eggs were negatively stained and visualized using electron microscopy (EM). Scale bar, 200 nm. (**B**) The WSN virions collected from embryonated chicken eggs were purified by sedimentation on a 30% sucrose cushion and subjected to Western blotting (left). Purified virions were further sedimented on a sucrose gradient (30 to 60%), and 12 fractions were collected from the top and analyzed with Western blotting (right). (**C**) The WSN virions collected from embryonated chicken eggs were purified by sedimentation on a 30% sucrose cushion, then the pellet was fixed, and avANP32A in viral particles were analyzed by immunogold labeling with anti-rabbit ANP32A antibody. Anti-rabbit IgG antibody was used as a negative control in our assays. Labeled samples were stained and visualized by EM. Scale bar, 100 nm. (**D**) WSN virions obtained from embryonated chicken eggs were purified and lysed for SDS-PAGE, and the part of the gel cropped by the frame was subjected to LC-MS/MS. Four avANP32A peptides (gray marked peptides) were identified in WSN virion samples. (**E**) Experiments were performed as in (B), except that the WSN virus amplified from HEK293T cells was purified by ultracentrifugation with a prior HAd step. (**F**) Experiments were performed as in (B), except that the WSN virus amplified from HEK293T cells overexpressing Flag-tagged avANP32A or with an empty vector was purified by ultracentrifugation with a prior HAd step. (**G**) MDCK cells were transfected with Flag-tagged avANP32A. After 24 hours, cells were infected with WSN (MOI = 10) for 7 hours and then fixed for IEM with mouse anti-IgG and mouse anti-Flag antibody. Arrows indicate avANP32A inside the IAV virions. Scale bar, 200 nm.

To determine whether ANP32 proteins are incorporated into IAV particles, influenza A/WSN/33 virus (WSN) produced from embryonated chicken eggs were purified by ultracentrifugation and the virions were checked by electron microscopy (Fig. 1A). An ANP32A polyclonal antibody that recognizes endogenous avANP32A (fig. S1) was used to verify the presence of endogenous avANP32A in the virions. As expected, avANP32A was specifically detected in WSN virion samples but not in mock samples (Fig. 1B, left). Here, the presence of β-actin (ACTB), one of the most abundant host proteins in the virions (*31*, *32*), in samples of lysed virions was used as a positive control in our assays. The purified WSN virions were then subsequently subjected to a sedimentation experiment using 30 to 60% sucrose density ultracentrifugation, and the avANP32A was shown to comigrate with the vRNP proteins of WSN virus (Fig. 1B, right). To further prove the presence of ANP32 within the influenza virions, immunoelectron microscopy (IEM) analysis was performed with the purified WSN virions produced from embryonated chicken eggs, and the results showed that endogenous avANP32A could be detected inside the virions (Fig. 1C and table S1). In addition, avANP32A was unambiguously identified in WSN virions using liquid chromatography–tandem mass spectrometry (LC-MS/MS) (Fig. 1D).

To further confirm this phenomenon, WSN viruses were produced from human embryonic kidney (HEK) 293T cells or HEK293T cells overexpressing Flag-tagged avANP32A and were purified by a prior hemadsorption/elution assay (HAd) (fig. S2A) to remove the contamination of extracellular vesicles (EVs), such as exosomes. The purification method used here, by introducing a first step of hemadsorption (HAd) to and elution from red blood cells selected for material with both receptor binding and cleavage activities [as provided by viral HA and neuraminidase (NA)], was shown to be effective in increasing the stringency of the purification of influenza virus as reported in previous reports (*31*, *33*, *34*). As shown in fig. S2 (B and C), the uninfected samples purified by ultracentrifugation without a prior HAd contained cluster of differentiation 81 (CD81) and ACTB proteins, which are two common markers for exosomes, suggesting that samples purified by ultracentrifugation are indeed contaminated with EVs. With a prior HAd step, the CD81 and ACTB proteins cannot be detected in the uninfected samples, which strongly suggested that the contamination of EVs can be completely removed by a prior HAd step. The CD81 and ACTB are also proven to be associated with IAV virion (*31*, *32*). Similarly, we found that CD81 and ACTB proteins were still detectable after the prior HAd step in samples infected with WSN virus, suggesting that the CD81 and ACTB are associated with IAV particles. Our results also showed that Flag-tagged avANP32A and endogenous huANP32A were detected in the uninfected samples without HAd, but not with HAd, in agreement with the fact that ANP32 proteins are a component of EVs (*35*). In addition, this finding further supports the idea that with a prior HAd step, the EVs can be efficiently removed. Flag-tagged avANP32A and endogenous huANP32A were detected in the WSN-infected samples even with a prior HAd (fig. S2, B and C, and Fig. 1, E and F), and as shown in Fig. 1 (E and F), Flag-tagged avANP32A and endogenous huANP32A comigrate with the vRNP proteins of WSN virus. These data again demonstrate that IAV particles can package the ANP32A protein.

To directly confirm the occurrence of the packaging process of avANP32A in IAV virions, Madin-Darby canine kidney (MDCK) cells overexpressing avANP32A-Flag were infected with WSN virus produced from embryonated chicken eggs at a multiplicity of infection (MOI) of 10 for 7 hours. IEM analysis was performed to verify the incorporation of avANP32A. Staining with control mouse immunoglobulin G (IgG) antibody showed only a background signal, whereas a clear avANP32A-Flag signal was detected in the virions following treatment with anti-Flag antibodies (Fig. 1G and table S2). Overall, these results indicated that ANP32A can be incorporated into WSN virions.

**Fig. 2.**
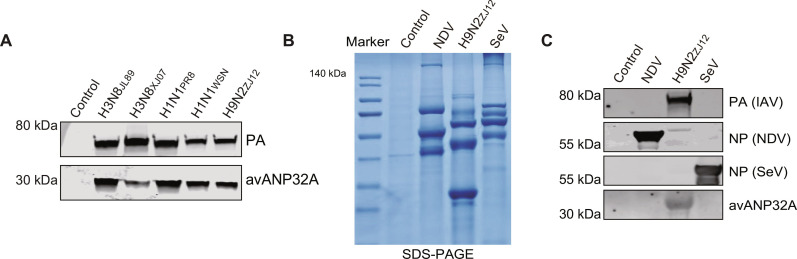
Packaging of ANP32 proteins is a general phenomenon for IAVs. (**A**) Lysates of purified influenza A virions from different species produced from embryonated chicken eggs were subjected to Western blot analysis. (**B** and **C**) NDV, H9N2, and SeV produced from embryonated chicken eggs were purified by sedimentation on a 30% sucrose cushion and lysed for SDS-PAGE (B) and Western blot analysis to detect avANP32A (C).

Host factor ANP32 proteins are essential for supporting the activity of IAV polymerases from different species (*18*–*20*). We found that WSN virus can incorporate both human and avian ANP32 proteins in the abovementioned experiments. We therefore speculated that the packaging of ANP32 proteins into IAV particles is a general phenomenon. To test this hypothesis, we purified IAV particles, produced in embryonated chicken eggs, from other taxa including humans, birds, and equines, and found that all the tested IAVs could package endogenous avANP32A into their own virus particles (Fig. 2A). To further prove that the package of endogenous avANP32A is specific to IAV when produced from embryonated chicken eggs, Sendai virus (SeV) and Newcastle disease virus (NDV) produced from embryonated chicken eggs were purified by using the same protocol. Similar amounts of proteins from different virions were subjected to SDS–polyacrylamide gel electrophoresis (SDS-PAGE) and Western blotting for analysis of the presence of avANP32A (Fig. 2, B and C). As shown in Fig. 2C, the presence of avANP32A was specially detected in the AIV (H9N2_ZJ12_), but not in the SeV or NDV samples. Overall, these results indicated that ANP32A can be incorporated into both mammalian-adapted influenza A virions and avian influenza A virions. Together, these results suggested that host factor ANP32 proteins could be packaged by the IAV particles.

### ANP32 proteins are incorporated into influenza B viral particles

**Fig. 3.**
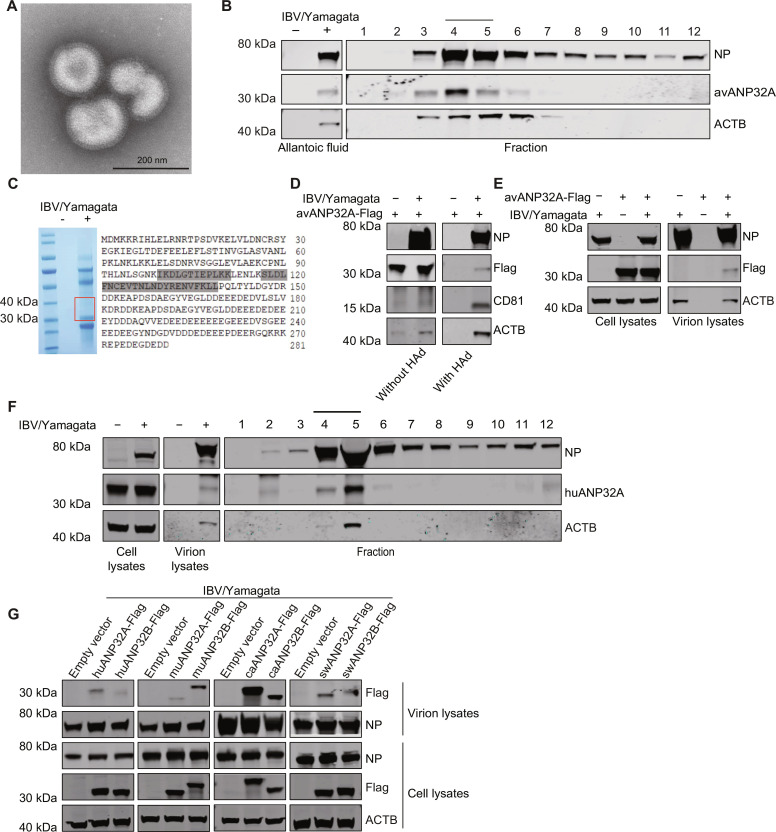
ANP32 proteins are packaged in IBV particles. (**A**) Purified IBV/Yamagata virions produced from embryonated chicken eggs were negatively stained and visualized using EM. Scale bar, 200 nm. (**B**) Experiments were performed as in Fig. 1B, except that the IBV/Yamagata virions amplified from embryonated chicken eggs were used. (**C**) IBV/Yamagata virions obtained from embryonated chicken eggs were purified and lysed for SDS-PAGE, and the part of the gel cropped by the frame was subjected to LC-MS/MS. Two avANP32A peptides (gray marked peptides) were identified in IBV/Yamagata virion samples. (**D**) HEK293T cells transfected with avANP32A-Flag constructs were infected with IBV/Yamagata virus (MOI = 0.01), and 48 hours later, the supernatants from mock-infected and IBV-infected cells were pelleted by ultracentrifugation either with or without a prior HAd step. Afterward, the pellets were resuspended and subjected to Western blot analysis. (**E**) HEK293T cells were transfected either with Flag-tagged avANP32A or with an empty vector. After 48 hours, cells were infected with IBV/Yamagata virus (MOI = 0.01) for further 48 hours. Medium was then collected for ultracentrifugation to purify the virus, with a prior HAd step. The cell lysates and purified virions were subjected to Western blot analysis. (**F**) Experiments were performed as in Fig. 1E, except that the IBV/Yamagata virus was used. (**G**) HEK293T cells were transfected with Flag-tagged ANP32 proteins from different species, and then transfected cells were infected with IBV/Yamagata virus (MOI = 0.01) for further 48 hours. Medium was then collected for virus purification by ultracentrifugation with a prior HAd step. The lysates of the purified virions were then analyzed with Western blotting. Hu, human; mu, mouse; ca, canine; sw, swine.

To determine whether IBV viral particles package ANP32 proteins, IBV/Yamagata virus produced from embryonated chicken eggs were purified by ultracentrifugation and the virions were checked by electron microscopy (Fig. 3A). Western blot analysis showed that avANP32A was specifically detected in IBV/Yamagata virion samples and comigrated with the NP protein of IBV/Yamagata virus (Fig. 3B). In addition, avANP32A was unambiguously identified in IBV/Yamagata virions using LC-MS/MS (Fig. 3C). To further confirm this phenomenon, IBV/Yamagata virions were produced from wild-type HEK293T cells or HEK293T cells overexpressing Flag-tagged avANP32A and followed by ultracentrifugation with a prior HAd assay. We found that CD81 and ACTB were eliminated in the uninfected samples with a prior HAd step. However, in IBV/Yamagata virus–infected samples, these two proteins can still be detected even with a prior HAd step (Fig. 3D), suggesting that the IBV virus could package CD81 and ACTB. After eliminating the contamination of EVs with a prior HAd step successfully, we confirmed that IBV/Yamagata virions could package overexpressed Flag-tagged avANP32A and endogenous huANP32A (Fig. 3, E and F). Moreover, we also purified IBV/Yamagata viruses from HEK293T cells overexpressing ANP32A/B proteins from different species, followed by Western blot analysis. The results indicate that both ANP32A protein and ANP32B protein from all the tested species can be incorporated into IBV/Yamagata virions (Fig. 3G). Taking these results together, we concluded that ANP32 proteins could be packaged by influenza B viral particles.

### ANP32A transferred by viral particles supports the next round of influenza virus replication in the target cells

**Fig. 4.**
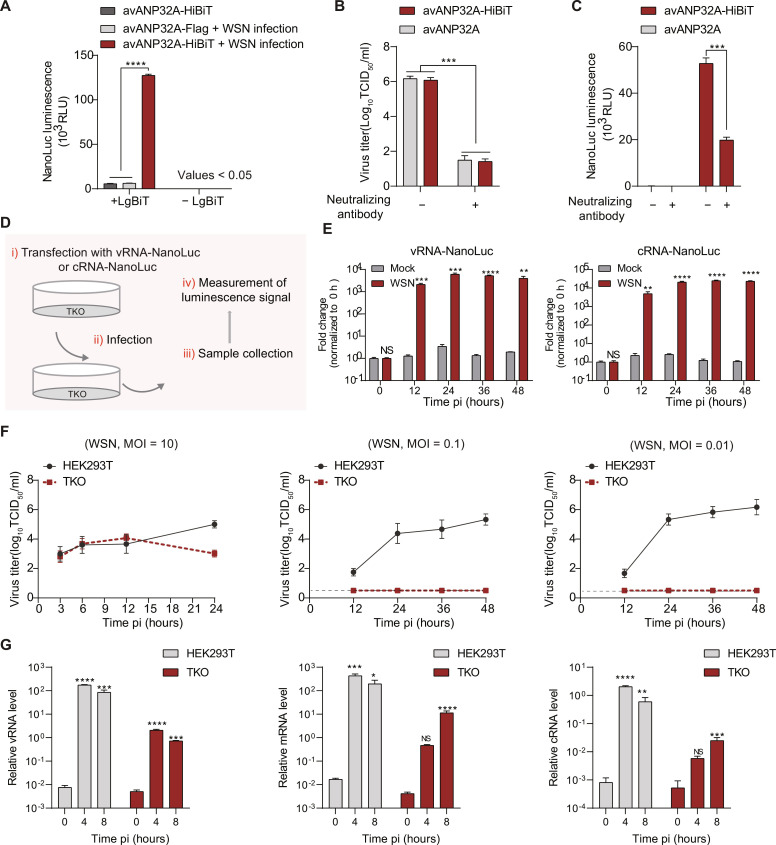
avANP32 transferred by viral particles supports influenza virus replication in target cells. (**A**) Detection of the amount of avANP32A-HiBiT in the target cells transferred by influenza viral particles upon virus infection by using NanoBiT technology. RLU indicates relative luminescence unit. (**B** and **C**) HEK293T cells expressing avANP32A and HiBiT-tagged avANP32A were infected with influenza IAV/H3N8_JL89_ (MOI = 0.01) for 48 hours before collection of medium for purification of virions with a prior HAd step. The purified virus was treated with neutralizing antibody, and then the infectivity was determined using standard methods in MDCK cells (B). The amount of avANP32A-HiBiT in MDCK cells infected with IAV/H3N8_JL89_ was detected using NanoBiT technology (C). (**D**) Schematic representation of the protocol for the experiments shown in (E). (**E**) Measurement of IAV polymerase activity in TKO cells transfected with vRNA-NanoLuc (left) and cRNA-NanoLuc (right) during IAV infection (MOI = 10). (**F**) Viral growth kinetics of WSN in HEK293T cells and TKO cells. The dashed line indicates the low limit of detection. (**G**) Levels of vRNA, cRNA, and mRNA of the viral NP segment were determined using quantitative reverse transcription PCR (RT-PCR) in HEK293T cells and TKO cells infected with WSN at an MOI of 5 for the indicated time points after infection. In (A) to (C) and (E) to (G), error bars represent mean ± SD from *n* = 3 independent biological replicates. NS (not significant), *P < 0.05,**P < 0.01, ***P < 0.001, and ****P < 0.0001 by unpaired *t* test (A to E) or one-way analysis of variance (ANOVA) followed by Dunnett’s multiple comparisons test (G).

To confirm that the ANP32 proteins packaged in influenza virions can be transferred into target cells upon viral infection, we first measured the amount of transferred avANP32A in the target cells upon virus infection using conventional methods (Western blot analysis and confocal analysis). We failed to detect avANP32A in target cells, possibly because the amounts of packaged avANP32A protein were below the detection limits of these methods. To overcome this, we used NanoLuc Binary Technology (NanoBiT) (*36*) to assess the amount of packaged avANP32A transferred by the influenza virus into the target cells. NanoBiT quantifies the amount of 11-residue HiBiT peptide-tagged proteins with high sensitivity when it forms a complex with supplied complementary large NanoLuc fragment (LgBiT). We added an HiBiT tag to the C terminus of avANP32A and produced WSN virus that packaged either HiBiT-tagged avANP32A or untagged avANP32A in HEK293T cells overexpressing either avANP32A-HiBiT or avANP32A, respectively. In addition, the medium from HEK293T cells overexpressing avANP32A-HiBiT without WSN infection was also collected for purification with a HAd step and was saved as negative control samples. The purified IAV virions or negative control samples were then used to infect MDCK cells for 2 hours, and the luciferase activity of cells was measured to show the amount of transferred avANP32A in the target cells (fig. S3). We observed that the intensity of NanoLuc luminescence was significantly higher in the samples infected with IAV virions packaged with avANP32A-HiBiT than that in those containing either negative control samples or samples infected with IAV virions packaged with untagged avANP32A (Fig. 4A). To further confirm that the luciferase activity is mediated by virus infection, a virus neutralization assay was conducted by using equine influenza A virus H3N8_JL89_ and its neutralizing antibody produced in our laboratory. As shown in Fig. 2A, H3N8_JL89_ virus could package avANP32A. H3N8_JL89_ viruses produced in HEK293T cells overexpressing either avANP32A-HiBiT or avANP32A were purified with a prior HAd assay and incubated with the neutralizing antibody at 37°C for 30 min, and then half of the sample was subjected to a median tissue culture infectious dose assay in MDCK cells for determination of neutralizing efficiency, and the other half was used to infect MDCK cells for 2 hours for measurement of the amount of HiBiT-tagged avANP32A transferred by influenza A virus. As shown in Fig. 4B, treatment with the neutralizing antibody significantly reduced virus infectivity. Correspondingly, the amount of transferred HiBiT-tagged avANP32A in the target cells after viral transfection was significantly reduced by neutralizing antibody treatment (Fig. 4C). These experiments further confirmed the specific transfer of ANP32 into target cells by influenza A viruses upon infection. Collectively, these data suggest that the packaged avANP32A in the influenza A virions can be transferred into target cells.

To test whether the transferred ANP32 proteins support the activity of vPol, human *ANP32A/B/E *triple-knockout (TKO) human embryonic kidney 293T (HEK293T) cells (fig. S4A) (*28*), which completely lost support for the polymerase activity of both WSN and IBV/Yamagata virus, were used to establish an ANP32-dependent mini-genome assay. We found that cotransfection of complementary RNA (cRNA)–NanoLuc luciferase reporter and RNP proteins from WSN into TKO cells did not result in any induction of NanoLuc luciferase. However, reconstitution of functional ANP32 proteins, including huANP32A, huANP32B, and avANP32A, into TKO cells enables the coexpressed RNP proteins to function efficiently and drive replication and transcription of the cRNA NanoLuc luciferase reporter robustly. When these experiments were performed in HEK293T cells, RNP proteins alone functioned well without reconstitution of ANP32 proteins (fig. S4B). These results suggest that an ANP32-dependent mini-genome assay established in TKO cells can be used to test whether transferred ANP32 proteins can support polymerase activity.

To this end, the TKO cells were transfected with a vRNA-NanoLuc luciferase reporter for 24 hours before infection with WSN at an MOI of 10 containing avANP32A produced from embryonated chicken eggs. The supernatant medium was then collected for the measurement of NanoLuc luminescence at different time points after infection (Fig. 4D). The vRNA-NanoLuc luciferase reporter is replicated and transcribed only by the influenza polymerase supported by ANP32 proteins in TKO cells. As shown in Fig. 4E (left), we observed robust induction of the NanoLuc luciferase during IAV infection, whereas only limited induction of NanoLuc luciferase was observed in mock samples. This implied that transferred avANP32A supports influenza polymerase activity. To investigate this issue further, a cRNA-NanoLuc luciferase reporter was used to rule out the effect of primary transcription of incoming vRNPs. We observed robust induction of cRNA-NanoLuc luciferase during IAV infection Fig. 4E (right). These results show that transferred avANP32A supports the replication of influenza polymerase. We made similar observations in the context of IBV/Yamagata virus (fig. S5, A and B). These data suggested that host factor ANP32 proteins delivered upon influenza virus infection were functional in supporting influenza polymerase activity for both IAV and IBV.

To directly assess the function of the transferred ANP32 proteins on virus replication, we infected HEK293T cells and TKO cells with WSN virus produced from embryonated chicken eggs at different MOI and determined viral titers at the indicated time points after infection. At low MOI, such as 0.1 and 0.01, no infectious progeny viruses were detected in TKO cells; however, at an MOI of 10, infectious progeny viruses produced in TKO cells were clearly observed (Fig. 4F). In addition, we made similar observations in the context of IBV/Yamagata virus (fig. S5C). These data suggested that a certain amount of transferred avANP32A could support viral replication in TKO cells. Meanwhile, we also measured the levels of the products of NP vRNA replication (vRNA and cRNA) and transcription (mRNA) in TKO cells and HEK293T cells infected with WSN virus at an MOI of 5. As shown in Fig. 4G, the levels of these three types of RNA in TKO cells significantly increased over time following virus infection. Together, these data suggest that the transferred ANP32A could be used for viral replication in the target cells.

**Fig. 5.**
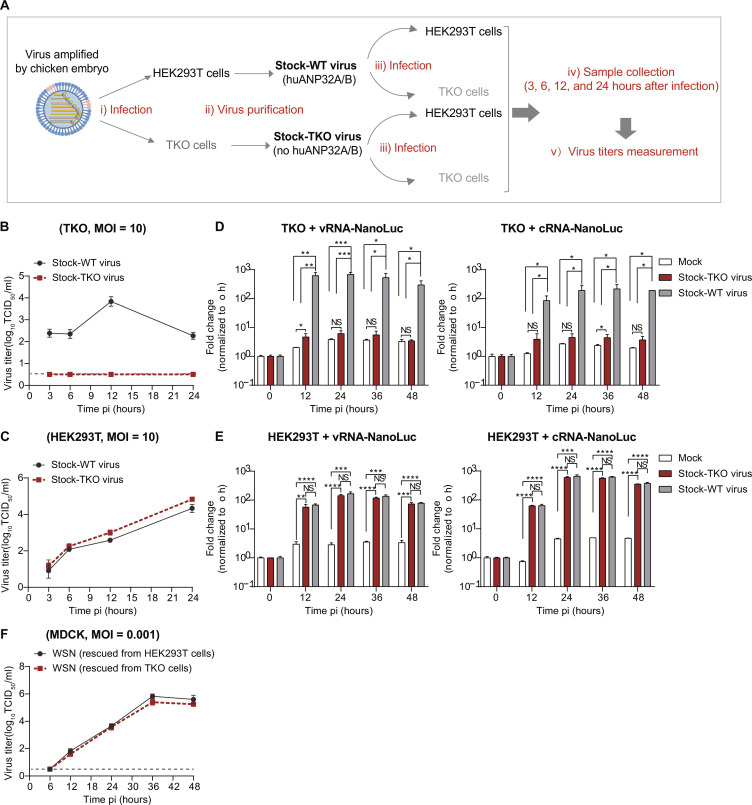
Human ANP32 transferred by viral particles supports IAV replication in target cells. (**A**) Schematic representation of the protocol for the experiments shown in (B) and (C). HEK293T cells and TKO cells were infected with WSN virus at an MOI of 10 for 24 hours. Medium was collected for virus purification by ultracentrifugation with a prior HAd step. The Stock-WT virus and Stock-TKO virus were subsequently tittered in MDCK cells, and the viruses were used to infect HEK293T and TKO cells separately. Viral titers were measured at the indicated time points after infection. (**B** and **C**) Stock-WT virus and Stock-TKO virus were tittered in MDCK cells and used to infect TKO (B) and HEK293T cells (C) separately. Viral titers were measured at indicated time points after infection. The dashed line indicates the low limit of detection. (**D** and **E**) Measurement of IAV polymerase activity in TKO cells (D) or HEK293T cells (E) transfected with vRNA-NanoLuc (left) and cRNA-NanoLuc (right) during IAV infection (MOI = 5). (**F**) Virus rescue was performed in HEK293T cells and TKO cells by transfection with plasmids encoding WSN viral proteins (PA/PB1/PB2/NP/HA/NA/M1/M2/NS2), together with plasmids expressing eight WSN vRNAs. The viruses rescued from these two different cell lines were then used to infect MDCK cells (MOI = 0.001) separately. The supernatants were collected at different time points after infection for the measurement of viral titers. The dashed line indicates the low limit of detection. In (B) to (F), error bars represent mean ± SEM from *n* = 3 independent biological replicates. NS, *P* > 0.05, *P < 0.05, **P < 0.01,***P < 0.001, and ****P < 0.0001 by unpaired *t* test.

To further investigate the role of the packaged ANP32A in viral replication in TKO cells, WSN was used to infect HEK293T or TKO cells at an MOI of 10. At 24 hours after infection, the virions produced from HEK293T cells (named Stock-WT virus) or TKO cells (named Stock-TKO virus) were harvested and purified using ultracentrifugation with a prior HAd step (Fig. 5A). The infectivity of the Stock-WT and Stock-TKO virus was further determined in HEK293T cells and TKO cells. We observed that in TKO cells, Stock-WT virus can replicate at a level similar to that in HEK293T cells, while the Stock-TKO virus showed no infectivity (Fig. 5B). However, the Stock-WT and Stock-TKO viruses had similar replication kinetics in HEK293T cells (Fig. 5C). To further confirm that the failure of productive infection of Stock-TKO virus in TKO cells is due to the amount of packaged ANP32 proteins being insufficient to support its polymerase activity, we performed polymerase activity assays in TKO cells transfected with a vRNA-NanoLuc luciferase reporter or a cRNA-NanoLuc luciferase reporter under viral infection conditions. As shown in Fig. 5D, infection with Stock-TKO virus results in a limited induction of NanoLuc luciferase in transfected TKO cells. At the same time, however, we observed that infection with Stock-WT virus resulted in a robust induction of NanoLuc luciferase in these transfected TKO cells. These results support the observation that Stock-TKO virus cannot establish productive infection in TKO cells at high MOI infection (Fig. 5B). Because Stock-TKO virus established productive infection in HEK293T cells, we speculated that the reason is that endogenous human ANP32A/B (huANP32A/B) supports viral polymerase activity. To confirm this, we performed similar experiments in HEK293T cells as described in Fig. 5D. We found that when HEK293T cells transfected with an IAV vRNA-NanoLuc luciferase reporter or IAV cRNA-NanoLuc luciferase reporter were infected with Stock-WT or Stock-TKO virus, both infection treatments resulted in robust induction of NanoLuc luciferase to a comparable extent (Fig. 5E), which supported the idea that Stock-TKO virus could establish productive infections in HEK293T cells (Fig. 5C). Together, these results suggest that packaged huANP32A/B proteins can be used by influenza virus to support their replication in TKO cells (target cells), and where no ANP32A/B is packaged in the virion, ANP32 proteins in the target cell can be used upon viral replication.

To test whether ANP32 proteins are indispensable for the process of assembly and release of influenza virus particles, WSN was rescued in TKO cells or HEK293T cells by cotransfecting a 17-plasmid system expressing eight WSN viral vRNAs and nine plasmids encoding WSN viral proteins (PA/PB1/PB2/NP/HA/NA/M1/M2/NS2) (*37*). The successful virus rescue in TKO cells demonstrated that ANP32 proteins were not required for the process of assembly and release of IAV particles (fig. S6). In addition, we found that viruses rescued from TKO and HEK293T cells had similar replication kinetics in MDCK cells (Fig. 5F), which further emphasized that the lack of the incorporation of ANP32 proteins in the virions did not affect virus replication in the next round of infection, where target cells had functional ANP32 proteins, which could support polymerase activity. Collectively, these data suggest that the influenza virus can use either virion-incorporated ANP32 proteins or the ANP32 protein in the target cells for the next round of infection.

### An ANP32-vPol interaction determines the specific packaging of ANP32 proteins into influenza viral particles

**Fig. 6.**
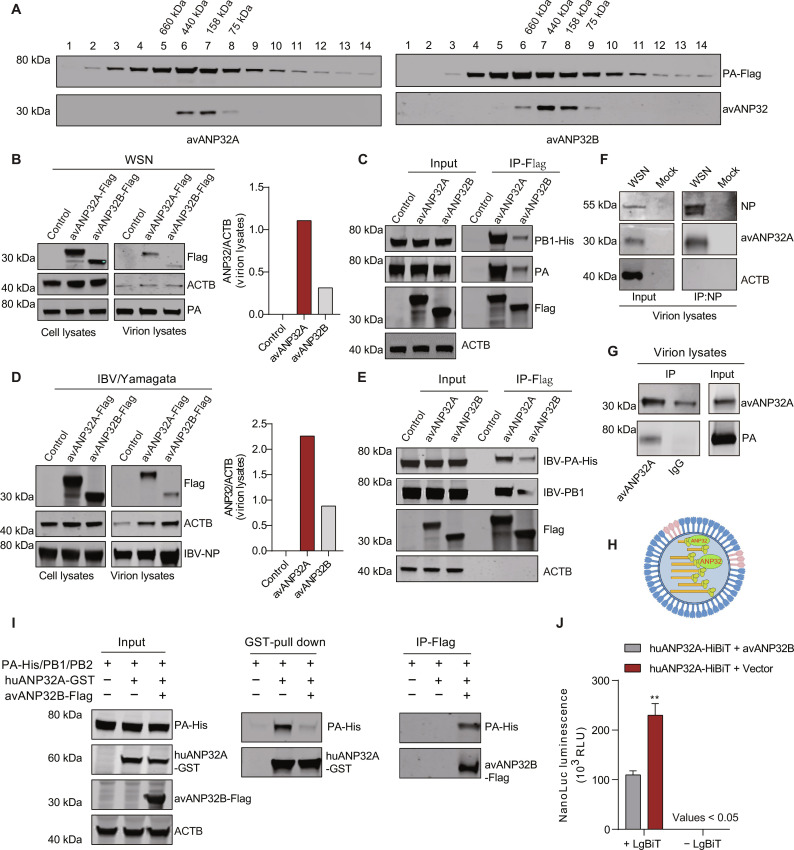
The ANP32-vPol interaction determines the packaging efficiency of ANP32 proteins in influenza viral particles. (**A**) HEK293T cells were transfected with PA-Flag, PB1, PB2 of WSN, and avANP32A/B-V5. The cell lysates were then immunoprecipitated with anti-Flag beads and then loaded onto a Superose 6 Increase 10/300 GL. The fractions obtained were analyzed using Western blotting with anti-Flag and anti-V5 antibodies. (**B**) Purified WSN virus produced from HEK293T cells overexpressing indicated plasmids, and cell lysates were subjected to Western blot analysis. The bar shows the relative intensity of packaged ANP32 proteins in WSN virus particles normalized to ACTB in purified virions from the left panel. (**C**) Co-IP experiments showing avANP32A interact much stronger with WSN-PB1/PA than that of avANP32B. (**D**) Experiment performed as in (B), except that IBV/Yamagata virus was used. (**E**) Co-IP experiments showing avANP32A interact stronger with IBV-PB1/PA than that of avANP32B. (**F**) WSN virions from embryonated chicken eggs were purified and lysed. Then, the vRNP was isolated from purified virions using anti-NP antibody and subjected to Western blot analysis to detect avANP32. (**G**) Purified WSN virions from embryonated chicken eggs were lysed and subjected to ANP32 antibody immunoprecipitation prior to Western blot analysis. (**H**) Model for the present form of ANP32 inside virions. (**I**) Co-IP experiments and GST pull down analysis showing that avANP32B overexpression suppressed huANP32A-PA interaction. (**J**) IBV/Yamagata virus produced from HEK293T cells overexpressing huANP32A-HiBiT with either avANP32B-Flag or an empty vector were purified using ultracentrifugation with a prior HAd step. Then, these purified viruses were subsequently used to infect MDCK cells at an MOI of 10 for 2 hours before lysis. The amount of huANP32A-HiBiT in MDCK cells was measured using NanoBiT technology. Error bars represent mean ± SD from *n* =3 independent biological replicates; unpaired *t* test; **P < 0.01.

As ANP32A or ANP32B can interact with vPol of IAV, IBV, and ICV (*18*, *20*, *28*, *30*), it is speculated that ANP32A or ANP32B is incorporated into influenza virions together with vPol. To test this hypothesis, we next investigated whether avANP32B could be packaged into influenza viral particles. avANP32B has lost its ability to support IAV polymerase activity due to the two amino acid mutations at sites 129 and 130 and showed weak interaction with vPol of IAV (*18*, *19*). We first intended to determine whether there is evidence for the existence of association between avANP32B and influenza virus polymerase. HEK293T cells were transfected with PA-Flag, PB1, PB2 of WSN, and avANP32A/B-V5. The intracellular polymerase complex was then purified using immunoprecipitation (IP) with anti-Flag beads and then loaded onto a Superose 6 Increase 10/300 GL. The analysis of the fraction eluted from the Superose 6 column indicates that avANP32A and a portion of polymerase coelute in fractions 6 to 8 and avANP32B and a portion of polymerase coelute in fractions 6 to 9 (Fig. 6A). These results suggest that, similar to avANP32A, avANP32B retains the ability to associate with vPol of IAV. Then, HEK293T cells overexpressing either avANP32A-Flag or avANP32B-Flag were infected with WSN. The virions in the medium were then purified and subjected to Western blot analysis. The avANP32B could be detected in the viral particles as expected (Fig. 6B). However, the amount of packaged avANP32B was much lower than that of avANP32A (Fig. 6B), which is consistent with the fact that the interaction between avANP32B and vPol was very weak compared with that between avANP32A and vPol in the cells (Fig. 6C). We observed similar results following infection of HEK293T cells with IBV/Yamagata virus, where we observed a positive correlation between the amount of packaged ANP32 protein and the binding affinity between ANP32 proteins and vPol (Fig. 6, D and E). On the basis of these findings, we speculated that the packaged ANP32A was likely to be associated with vPol in the context of vRNP inside the virions. To gain more evidence to support this view, the purified WSN virions produced from embryonated chicken eggs were lysed for IP with anti-NP antibodies. As shown in Fig. 6F, avANP32A, not ACTB, could be coimmunoprecipitated by NP, suggesting that avANP32A was associated with vRNP in the virions. To further prove our results, we also performed IP assay with anti-avANP32A antibodies, and results found that the PA subunit could be coimmunoprecipitated by anti-avANP32A antibodies (Fig. 6G). Together, these results suggested that the avANP32A was associated with vRNP in the viral particles (Fig. 6H).

Because the binding affinity between ANP32 proteins and vPol determines the amount of ANP32 proteins packaged in the influenza virus, any changes in the binding affinity should be reflected by the amount of packaged ANP32 proteins. Overexpression of avANP32B was found to inhibit the interaction between huANP32A and IBV/Yamagata polymerase (Fig. 6I), and the amount of transferred huANP32A-HiBiT was greatly reduced in the presence of avANP32B (Fig. 6J), indicating a competitive effect of avANP32B during incorporation. Overall, these results suggest that host factor ANP32 proteins are packaged into virus particles through interacting with vPol and that the ability of ANP32 proteins to bind to vPol determines the efficiency with which they are packaged.

### Avian ANP32A transferred by avian influenza A virus benefits the process of obtaining adaptive mutations

**Fig. 7.**
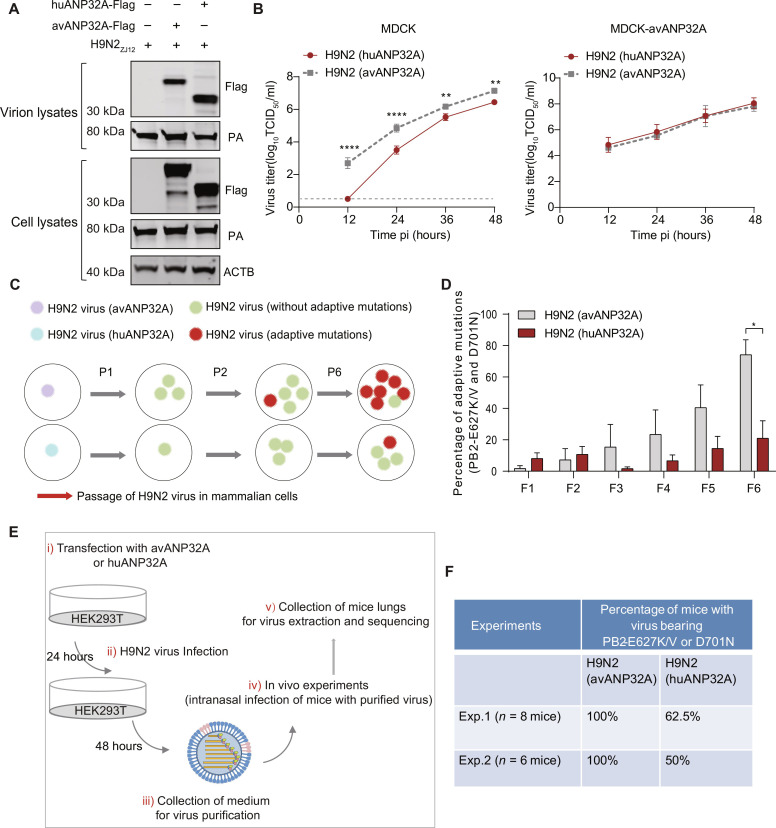
avANP32A transferred by avian influenza A virus accelerates the process of obtaining adaptive mutations. (**A**) H9N2 virus produced from HEK293T cells overexpressing Flag-tagged ANP32A or with an empty vector were purified by ultracentrifugation with a prior HAd step. The cell lysates and purified virions were then subjected to Western blotting. (**B**) Viral replication in MDCK cells or MDCK-avANP32A cells infected with H9N2 (huANP32A) virus and H9N2 (avANP32A) virus at an MOI of 0.1. Error bars represent mean ± SD from *n* = 3 independent biological replicates; unpaired *t* test; **P < 0.01 and ****P < 0.0001. (**C**) Model for the effect of avANP32A transferred by avian influenza A virus on viral replication and adaptive mutation acquisition when jumping from avian hosts to mammalian hosts. (**D**) H9N2 virus packaged with either avANP32A or huANP32A was blind passaged six times in MDCK cells. Viral RNAs were extracted and the C terminus of PB2 was amplified and deep-sequenced to monitor the residue phenotype of PB2 627 and 701 positions during passages in MDCK cells. Bar graph represents the percentage of adaptive mutations including PB2-E627K/V and D701N in each passage. Error bars represent mean ± SEM from *n* = 4 independent biological replicates, unpaired *t* test; *P < 0.05. (**E**) Schematic representation of the protocol for the experiments shown in (F). (**F**) The lungs of infected mice were collected for viral RNA extraction, and the C terminus of PB2 was amplified and cloned into T vectors. Nine molecular clones from each sample were randomly picked and sequenced for determination of the residue phenotype of PB2 627 and 701 positions. Samples with nine clone negatives for the emergence of PB2-E627K/V and D701N were recognized as no occurrence of adaptive mutations. In each group, the percentage of mice with emergence of adaptive mutations was calculated.

The species-specific differences in host factor ANP32 proteins determine the restriction of AIV vPol activity in mammalian cells (*15*). When AIV spreads across to mammals including humans, the AIV polymerase activity is poorly supported by huANP32A/B, and AIV therefore requires adaptive mutations such as PB2-E627K and PB2-D701N to overcome this limitation. Our results above suggest that ANP32 proteins from producer cells, including avANP32A, can be incorporated by multiple subtypes of influenza A virions, which includes H9N2 AIV virions, and can support the next round of viral replication. Therefore, we speculate that the avANP32A carried by AIV particles can enhance the replication of AIV in mammalian cells. To verify this hypothesis, H9N2 AIVs packaging either huANP32A (H9N2-huANP32A) or avANP32A (H9N2-avANP32A) were purified using HAd assay from HEK293T cells overexpressing either huANP32A or avANP32A. The Western blot analysis confirmed the successful package of either huANP32A or avANP32A in H9N2 virions (Fig. 7A). The viral titers were determined in the MDCK cells stably expressing Flag-tagged avANP32A and then used to infect MDCK cells at an MOI of 0.1, and the supernatants were collected over time for further measurement of virus replication. As shown in Fig. 7B, the replication efficiency of the H9N2-avANP32A virus in MDCK cells was significantly higher than that of the H9N2-huANP32A virus, indicating that avANP32A transferred by the virus particles indeed promotes the replication of the H9N2 virus in mammalian cells. The replication kinetics and titer of the H9N2-avANP32A virus in MDCK-avANP32A (stably expressing avANP32A) were comparable to those of the H9N2-huANP32A virus. These results suggest that the packaged avANP32A in AIV virions selectively prime AIV replication when they jump into mammalian cells.

Mammalian ANP32 proteins cannot efficiently support AIV vPol activity (*15*, *18*, *25*), and AIVs therefore need to obtain adaptive mutations. Given that the avANP32A packaged by nonadapted AIV primes early viral replication, we hypothesize that this replication advantage might benefit the initial interspecies transmission event by increasing the total number of viruses that have acquired adaptive mutations (Fig. 7C). To verify this hypothesis, four populations of H9N2-avANP32A virus and H9N2-huANP32A virus were passaged six times through MDCK cells, respectively. The eight gene segments from each population were Sanger-sequenced at passage 6, and the results revealed that two out of four (50%) populations of H9N2-avANP32A virus, but not that of H9N2-huANP32A virus, had acquired PB2-E627K/D701N mutations. However, mutations were not detected in seven other gene segments from these populations (table S3). To further quantify the frequency of the adaptive mutations in PB2 627 and 701 positions, the C terminus of PB2 from each population was deep-sequenced at passages 1 to 6, and the results found that the frequency of the previously identified adaptive mutations including PB2 E627K/V and D701N in the H9N2-avANP32A virus was significantly higher than that in the H9N2-huANP32A virus at passage 6 (Fig. 7D). To further confirm that the observed adaptive mutations in PB2 are the key dominant adaptive mutations in the experimental evolution approach with H9N2 virus, each population at passage 6 was further passaged for three more generations. The C terminus of PB2 from each population was Sanger-sequenced at passage 9. We found that all the populations of H9N2-avANP32A virus had evolved PB2-E627K/V or D701N, and two out of four (50%) populations of H9N2-huANP32A virus had acquired B2-E627K/V or D701N (table S3). Overall, these results suggest that during the experimental evolution of H9N2 avian influenza A virus in MDCK cells, packaged avANP32A in virions promotes H9N2 virus replication at the early stage of infection, and this replication advantage conferred by packaged avANP32 in virions increases the overall amount of virus with the acquisition of adaptive mutations.

To further clarify the role of packaged avANP32 in cross-species transmission of AIV, we determined the residue phenotype of PB2 627 and 701 for these two different stocks of H9N2 virus during infection in mice. Two experiments were performed. Mice were inoculated with either one of the H9N2 viruses, at an infectious dose of either 10^6.4^ 50% egg infection dose (EID_50_) (experiment 1) or 10^5.7^ EID_50_ (experiment 2). The lungs of mice were collected on the second day following high-dose infection and on day 4 following low-dose infection (Fig. 7E). vRNAs were extracted, and the C terminus of PB2 was amplified and cloned into T vectors. Nine molecular clones from each sample were randomly picked and sequenced for determination of the residue phenotype of PB2 627 and 701 positions. Samples with nine clone negatives for the emergence of PB2-E627K/V and D701N were recognized as no occurrence of adaptive mutations. In each group, the percentage of mice with emergence of adaptive mutations was calculated. On the basis of these two independent in vivo studies, we found that the number of mice with viruses bearing previously identified adaptive mutations, which include PB2-E627K/V and PB2-D701N, was higher when they had been challenged with the H9N2-avANP32A virus than with the H9N2-huANP32A virus (Fig. 7F). Together, these results suggest that avANP32A incorporated into avian influenza A virions may promote avian influenza A virus replication in mammals and further benefit the downstream interspecies transmission event.

## DISCUSSION

**Fig. 8.**
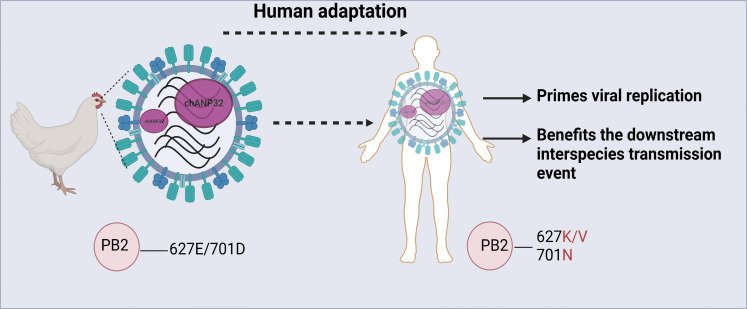
Schematic model of enhanced adaptation of AIV toward mammals by avANP32A packaged in viral particles. AIV packages avANP32A through its association with vPol in the context of vRNP when replicated in avian hosts. The packaged avANP32A will be released into target cells and selectively promotes the AIV vPol activity with AIV jumping into mammals. Enhanced replication in mammalian cells favors the adaptation of nonadapted AIV to mammals by increasing the total amount of virus with the acquisition of adaptive mutations. This figure was created by BioRender.

Host factor ANP32 proteins have been identified as fundamental cofactors of the influenza viral polymerase, and huANP32 proteins are key host factors responsible for the restriction of avian-signature polymerases in human cells (*15*, *18*–*20*, *23*–*25*). The well-known mammalian adaptive mutations, such as PB2-E627K, enable AIV vPol to efficiently use mammalian ANP32 proteins when AIVs jump from avian hosts to mammalian hosts (*15*, *18*). Here, we report that ANP32 proteins are incorporated into influenza viral particles by interaction with vPol and are transferred into newly infected cells upon infection to support virus replication. Furthermore, the amount of ANP32 protein packaged by the influenza virus is determined by the affinity of that ANP32 with vPol. avANP32A packaged in avian influenza A virions promotes early virus replication in mammalian hosts and further benefits the downstream interspecies transmission event (Fig. 8). Our results revealed a role and significance of avANP32A incorporated into virus particles in the cross-species transmission and adaptation of AIV.

All viruses, including influenza viruses, require host factors to complete their replication cycles (*38*). For influenza viruses, ANP32 proteins are vital host factors of the influenza viral polymerase (*15*, *17*–*20*, *39*, *40*). Differences between avian and mammalian ANP32 proteins underlie the host range barrier (*15*). avANP32A supports both avian and human-adapted influenza polymerases, whereas huANP32A/B only supports the latter because of the lack of the avian-specific 33–amino acid insert (*15*, *18*, *25*). However, the specific molecular mechanism by which ANP32 supports influenza viral polymerase remains unknown. Several studies have found that the function of ANP32 in supporting influenza polymerase is related to its interaction with vPol and that this interaction is enhanced in the context of vRNP (*15*, *18*, *23*–*25*, *29*), raising the possibility that ANP32 proteins are incorporated into influenza virus particles through an interaction with vPol. We tested this by purifying influenza virus particles to analyze the proteins inside the virions using Western blot and MS analysis. This study represents the first evidence that influenza viruses package ANP32 proteins. Previous failures to detect ANP32 proteins in influenza viral particles are probably due to the very low amounts present, which are further evidenced by our IEM analysis. The amount of ANP32 proteins packaged in influenza viral particles was sufficient to support viral replication in high-MOI single-cycle infections. However, it has only a limited effect on viral replication in low-MOI multicycle infections. These results are consistent with previous reports that influenza virus can replicate to a certain extent in chicken cells lacking ANP32A when infected at higher MOI (*19*). This is likely to be possible due to the characteristics of ANP32 proteins, namely, their long half-lives (*41*) and the very low amount of ANP32 proteins required to support polymerase activity (*18*). Host factor ANP32 proteins are mainly located in the nucleus (*20*, *25*, *42*) and are therefore unlikely to be packaged by accident into virus particles where influenza virion particle assembly takes place. We believe that this process is mediated by the strong interaction of ANP32 with vPol in the nucleus and that the ANP32 proteins are then selectively packaged into the virus particles through their binding with vPol. We demonstrated that the packaging of ANP32 by viral particles is independent of its support of viral polymerase activity and is determined instead by its affinity with vPol. Furthermore, recent studies showed that ANP32 is capable of mediating the formation of the viral polymerase complex, providing further structural evidence for the importance of ANP32 for the functioning of the polymerase complex (*30*). In addition, nonfunctional avANP32B can compete with functional huANP32A to bind with vPol, resulting in a reduction in the amount of huANP32A packaged in viral particles. Therefore, the role that functional ANP32 proteins packaged in viral particles have in supporting viral replication can be reduced through nonfunctional ANP32 proteins packaged by the virus. This could be an interesting idea for the treatment of avian influenza and could potentially reduce the threat of avian influenza to humans. Future work should explore the molecular basis underlying the packaging of ANP32 proteins in influenza virions and provide a theoretical basis for the further development of anti-influenza drugs.

Although ANP32 proteins have been confirmed to function in the replication of the viral genome (*15*, *17*–*20*), a large number of questions about the function of these proteins remain unanswered. Here, we found that the packaging of ANP32 is not necessary for viral particle assembly and release of WSN virus. The absence of ANP32 inside viral particles does not affect virus replication when infecting target cells at low MOI, because endogenous ANP32 proteins in the target cells can be used to support polymerase activity.

A previous study showed that AIVs with low polymerase activity could easily acquire adaptive mutation when infected by mammalian hosts (*43*). It is a question of how adaptive mutations could occur when AIVs infect mammalian cells. The successful acquisition of adaptive mutations suggests that although mammalian ANP32A/B cannot effectively support the replication of AIV, the virus could replicate to a certain level by unknown methods before adaptive mutations are acquired. The role of the PB2 E627K and D701N mutations in mammalian adaptation has been confirmed in a variety of AIVs, including the H5N1, H7N9, and H9N2 subtypes (*5*, *44*–*46*). It is not known whether AIVs use other mechanisms to adapt to mammalian cells during cross-species transmission, although we recently found that the viral protein NS2 relies on its SUMO-interacting motif to promote AIV adaptation to mammals by promoting mammalian ANP32A/B-supported avian vPol activity (*47*).

Here, we used the low-pathogenicity AIV subtype H9N2, which easily acquires the PB2 E627K and D701N mutations when it infects mammalian cells (*43*, *45*), as a model to evaluate the impact of transferred avANP32A on the AIV replication and acquisition of adaptive mutations by the AIV in mammalian hosts. We demonstrate that avANP32A transferred by AIV can enhance the replication of AIV under unfavorable conditions in mammalian cells. This efficient supportive function for the avian vPol activity by avANP32A transferred in viral particles favors the acquisition of adaptive mutations in the viral polymerase, thereby enhancing the ability of the AIV to jump to mammals. Our results further illustrate that in the process of obtaining adaptive mutations driven by the low polymerase activity, the level of viral replication before the acquisition of adaptive mutations affects the downstream interspecies transmission event.

To conclude, our study suggests that the host factor ANP32A incorporated into virus particles has a significant role in the support of viral replication in newly infected cells, especially in the process of interspecies infection of AIV. We identified a previously unknown strategy by which AIVs package avANP32A into their viral particles to assist in the crossing of the species barrier from birds to mammals.

## MATERIALS AND METHODS

### Ethics statement

The 9-day-old embryonated chicken eggs and chicken blood were obtained from the National Poultry Laboratory Animal Resource Center. This study was conducted in strict accordance with the recommendations in the *Guide for the Care and Use of Laboratory Animals* of the Ministry of Science and Technology of the People’s Republic of China. Animal experiments were approved by the Committee on the Ethics of Animal Experiments of the Harbin Veterinary Research Institute of the Chinese Academy of Agricultural Sciences (CAAS).

### Cell lines and culture conditions

HEK293T, chicken embryonic fibroblasts cells (DF1), and MDCK cells were maintained in Dulbecco’s modified Eagle’s medium (DMEM; Sigma-Aldrich) supplemented with 10% fetal bovine serum and 1% penicillin/streptomycin. The generation of the ANP32A, ANP32B, and ANP32E TKO HEK293T cell lines (TKO) has been described previously (*18*, *28*). MDCK cells stably expressing Flag-tagged avANP32A were generated previously (*47*). All cells were maintained at 37°C in a 5% CO_2_ atmosphere.

### Virus and plasmids

The H1N1 human IAV A/WSN/1933 (WSN) was provided by Y. Kawoaka; the H3N8 equine IAV A/equine/Jilin/1/1989 (H3N8_JL89_) and the H3N8 equine IAV A/equine/Xinjiang/1/2007 (H3N8_XJ07_) were preserved in our laboratory; the H9N2 avian IAV A/chicken/Zhejiang/B2013/2012 (H9N2_ZJ12_) was provided by Z. Li from the Shanghai Veterinary Research Institute; and the IBV B/Yamagata/PJ/2018 was preserved in our laboratory and has been described previously (*28*). The eight reverse genetics plasmids for the rescue of the A/PR/8/34 (PR8) virus were provided by R. Fouchier from Erasmus MC, Rotterdam, The Netherlands.

The pCAGGS plasmids encoding ANP32A/B from different species have been described previously (*18*, *27*, *28*). The plasmids encoding the genes of IBV B/Yamagata/1/73 were provided by Y. Kawoaka. The pCAGGS plasmids encoding ANP32A/B proteins with V5 tags or HiBiT tags and the plasmids expressing the genes from the different influenza viruses mentioned above were generated using overlapping polymerase chain reaction (PCR) and were identified using DNA sequencing.

### Western blotting and antibodies

Virus lysates and cell lysates were subjected to Western blot analysis using standard protocols as described previously (*18*). Immunoblotting analysis was carried out using the following primary antibodies: ANP32A rabbit polyclonal (15810-1-AP, Proteintech), ANP32B mouse monoclonal (66160-1-Ig, Proteintech), ANP32E rabbit polyclonal (A17220, Abclonal), Flag mouse monoclonal (F1804, Sigma-Aldrich), Flag rabbit polyclonal (F7425, Sigma-Aldrich), HA mouse monoclonal (H9658, Sigma-Aldrich), ACTB rabbit monoclonal (AC026, Abclonal), His mouse monoclonal (66005-1, Proteintech), V5 mouse monoclonal (ab27671, Abcam), glutathione *S*-transferase (GST) rabbit polyclonal (10000-0-AP, Proteintech), CD81 mouse monoclonal (66866-1-Ig, Proteintech), and IBV NP rabbit polyclonal (GTX128538, GeneTex). Anti–IAV-PB2, anti–IAV-PA, and anti–IBV-PB1 are mouse monoclonal antibodies generated in our laboratory.

### Purification of virus particles

Virus particles were purified as previously described (*31*). Briefly, allantoic fluid harvested from infected eggs was cleared by centrifugation (10,000 rpm, 20 min, 4°C), and the supernatant was concentrated through a 30% sucrose cushion in sodium tris (hydroxymethyl)-aminomethane (NT) buffer containing ethylenediaminetetraacetic acid (EDTA) (NTE) buffer [100 mM NaCl, 1 mM EDTA, and 10 mM HCl-Tris (pH 7.5)] (100,000*g*, 2 hours, 4°C) in an SW28Ti rotor (Beckman Coulter). The pellet was resuspended in NTE buffer and centrifuged over a 30 to 55% sucrose-NTE step gradient (100,000*g*, 2 hours, 4°C) in an SW41Ti rotor (Beckman Coulter). The visible band in the 30 to 50% interface was collected and diluted in the same buffer, followed by concentration at 100,000*g* for 2 hours. For gradient fractionation, the virus pellet was centrifuged over a noncontinuous 30 to 60% sucrose gradient. Twelve fractions were collected from the top followed by concentration at 100,000*g* for 2 hours. The pellet was resuspended in 50 μl of phosphate-buffered saline (PBS) and analyzed with Western blotting. Virus growing in infected HEK293T cells were purified with the more stringent HAd technique to eliminate contamination of EVs. Briefly, growth medium containing infected cells was cleared by filtering through a 0.2-μm filter (Millipore) and then mixed with adult chicken blood (20%) at a final packed cell volume of approximately 0.2%. The suspensions were kept at 4°C for 30 min, shaking gently with an orbital shaker, after which the blood cells were pelleted (1350*g* for 5 min at 4°C) and washed twice with chilled PBS. The cell pellet was then resuspended in 37°C PBS and incubated at 37°C for 30 min under constant gentle shaking. Last, the cells were pelleted (5000*g* for 5 min at 25°C), and the supernatant was concentrated by centrifugation at 28,000 rpm at 4°C for 2.5 hours layered onto a 30% sucrose cushion, after which purification proceeded as above.

### Electron microscopy

For negative staining, the purified IAV and IBV virion samples were fixed with 2.5% glutaraldehyde (pH 7.2) overnight. A 20-μl drop of sample was applied to a carbon-coated grid that had been glow-discharged for 20 s in air, and the grids were immediately negatively stained using 2% phosphotungstic acid. Grids were examined in an H-7650 (Hitachi, Tokyo, Japan) operated at 80 kV.

For the IEM, MDCK cells overexpressing avANP32A-Flag were infected with WSN at an MOI of 10. At 7 hours after infection, the cell layer was carefully removed and centrifuged (4000 rpm, 15 min, room temperature). The supernatant was removed and the cells were fixed in 0.1 M HEPES containing 4% paraformaldehyde and 0.1% glutaraldehyde (2 hours, 4°C). Gradient dehydration was performed with (50, 70, 90, and 100%) *N*,*N*′-dimethylformamide for 15 min at 4°C. Samples were then embedded in resin overnight at room temperature and polymerized under ultraviolet light (10 days, −20°C). Ultrathin sections (70 nm) were obtained in a Leica–EM-UC6 ultramicrotome and collected on formvar-coated nickel grids. Cell sections were blocked in 3% bovine serum albumin (BSA) (30 min at room temperature) and then incubated with anti-Flag mouse (1:50) or anti-IgG mouse antibodies (1:50; 40 min at room temperature). After washing in distilled water, grids were incubated with a 10-nm colloidal gold-conjugated goat anti-mouse IgG antibody (1:50; 40 min), washed in water, dried, and contrasted with saturated uranyl acetate (10 min at room temperature). Samples were analyzed on an H-7650 (Hitachi, Tokyo, Japan) operated at 80 kV.

For the IEM of WSN virus, allantoic fluid harvested from eggs was concentrated through a 30% sucrose cushion in NTE buffer in an SW28Ti rotor (Beckman Coulter). The pellet was removed and the IEM assay was performed with huANP32A antibody (1:50) and anti-rabbit IgG antibody (1:50).

### IP assay

Transfected cells were lysed in lysis buffer [50 mM Hepes-NaOH (pH 7.9), 100 mM NaCl, 50 mM KCl, 0.25% NP-40, and 1 mM dithiothreitol] for 10 min on ice, and then cell debris was removed by centrifuging at 13,000*g* and 4°C for 10 min. The supernatant was incubated with anti-Flag M2 magnetic beads (Sigma-Aldrich, M8823)/GST beads (GE Healthcare) at 4°C for 2 hours or overnight. Following extensive washing with lysis buffer, bound proteins were eluted either with boiling in SDS-PAGE loading buffer or with 3× Flag peptides (Sigma-Aldrich).

### Quantitation of vRNAs in infected cells using RT-PCR

Total RNA from infected cells was extracted using an RNeasy mini kit (Tiangen) according to the manufacturer’s instructions. Complementary DNAs (cDNAs) complementary to the three types of WSN NP RNA were synthesized with tagged primers to add an 18- to 20-nucleotide adapter that was unrelated to the influenza virus (*48*). Specific primers were used in the reverse transcription reaction, as follows: NP vRNA, 5′-GGCCGTCATGGTGGCGAATGAATGGACGGAGAACAAGGATTGC-3′; NP cRNA, 5′-GCTAGCTTCAGCTAGGCATCAGTAGAAACAAGGGTATTTTTCTTT-3′; and NP mRNA, 5′-CCAGATCGTTCGAGTCGTTTTTTTTTTTTTTTTTCTTTAATTGTC-3′. The reverse transcription reaction was performed with the hot-start protocol using Superscript III Reverse Transcriptase (Invitrogen) as described previously (*17*). The cDNA samples were subjected to quantification with real-time PCR using SYBR Green (TAKARA) according to the recommended protocol. The primers used in the real-time PCR were as follows: vRNA F, 5′-GGCCGTCATGGTGGCGAAT-3′; vRNA R, 5′-CTCAATATGAGTGCAGACCGTGCT-3′; cRNA F, 5′-CGATCGTGCCCTCCTTTG-3′; cRNA R, 5′-GCTAGCTTCAGCTAGGCATC-3′; mRNA F, 5′-CGATCGTGCCCTCCTTTG-3′; and mRNA R, 5′-CCAGATCGTTCGAGTCGT-3′.

### Influenza virus infection

Influenza virus was rescued from a 12-plasmid rescue system and a 17-plasmid rescue system (*37*). For the 12-plasmid rescue system, HEK293T cells cultured in a 10-cm plate were transfected with 1 μg each of eight pPolI plasmids and 2 μg each of pCAGGS-NP, pCAGGS-PA, pCAGGS-PB1, and pCAGGS-PB2 using polyethylenimine(PEI). The medium was harvested after 48 hours, and the virus was propagated in specific pathogen–free embryonated eggs as the virus stocks were used to infect cells in this study. The cells were washed twice with PBS and incubated with the virus for 2 hours. Cells were then cultured at 37°C in DMEM containing 0.3% BSA and tosylsulfonyl phenylalanyl chloromethyl ketone (TPCK)–trypsin (Sigma-Aldrich) at 0.5 μg/ml for 293T or 2 μg/ml for MDCK. At the indicated time points, the culture supernatant was harvested, and the viral titers were determined using standard methods in MDCK cells (or MDCK cells stably expressing avANP32A for H9N2 virus) as previously described (*24*).

For the 17-plasmid rescue system for the WSN virus, HEK293T/TKO cells cultured in a 75-cm^2^ flask were transfected with 2 μg each of pPolI plasmids; 2 μg each of pCAGGS-PB2, pCAGGS-PB1, pCAGGS-HA, pCAGGS-NP, and pCAGGS-NA; 0.2 μg of pCAGGS-PA; 4 μg of pCAGGS-M1; 0.06 μg of pCAGGS-M2; and 0.6 μg of pCAGGS-NS2 using PEI. The medium was harvested after 48 hours, and plaque assays were performed on the MDCK cells. Briefly, MDCK cells cultured in 12-well plates were infected with 10-fold serial dilutions of viral supernatants in 1× DMEM (0.3% BSA) for 1 hour at 37°C. Then, the cells were washed with PBS and overlaid with 1% Seaplaque agarose (Lonza) in 1× DMEM [0.3% BSA and TPCK-treated trypsin (2 μg/ml)]. After 48 to 72 hours, the cells were fixed with formalin, and plaques were stained with 0.1% crystal violet and counted.

### Measurement of transferred ANP32 proteins upon virus infection using NanoBiT

HEK293T cells overexpressing avANP32A-HiBiT or avANP32A were infected with WSN. After 48 hours, the virus particles were purified from the growth medium of the infected cells as described above. Then, MDCK cells cultured in 24-well plates were then incubated with the virus packaging with either avANP32A-HiBiT or avANP32A at an MOI of 5 for 2 hours before lysis. The amount of HiBiT tagged avANP32A in MDCK cells was then assessed using a Nano-Glo HiBiT Lytic Detection system according to the manufacturer’s instructions.

### Polymerase activity assay

To measure the polymerase activity assay in TKO cells or HEK293T cells infected with influenza virus, cells were transfected with the vRNA-luciferase reporter plasmid, pPolI-WSN-HA-untranslated regions (utr)-vRNA-Nanoluc/pPolI-IBV/Yamagata/1/73-NSutr-vRNA-Nanoluc (negative-sense viral-like NanoLuc luciferase reporter) or pPolI-WSN-HAutr-cRNA-Nanoluc/pPolI-IBV/Yamagata/1/73-NSutr-cRNA-Nanoluc (positive-sense viral-like firefly luciferase reporter). Twenty-four hours after transfection, cells were infected with influenza virus. The supernatants were collected at indicated time points for measurement of the luciferase activity using the NanoGlo luciferase assay system (Promega).

To determine the effect of ANP32 proteins on the polymerase activity, HEK293T cells or TKO cells in 24-well plates were transfected with plasmids carrying viral proteins PB1 (80 ng), PB2 (80 ng), PA (40 ng), and NP (160 ng), together with 80 ng of pPolI-WSN-HAutr-cRNA-Nanoluc and 20 ng of plasmids encoding different ANP32 proteins as indicated, using PEI according to the manufacturer’s instructions. Cells were lysed with 100 μl of passive lysis buffer (Promega) at 24 hours after transfection, and luciferase activity was measured using the NanoGlo luciferase assay system (Promega).

### MS analysis

Samples were separated using SDS-PAGE and stained with Coomassie, followed by in-gel digestion. Briefly, the gel bands were manually excised. After destaining, reduction, and alkylation of cysteines, each of the protein bands was individually digested with sequence-grade trypsin for 16 hours at 37°C. The peptides extracted from the gel slices were concentrated using vacuum centrifugation. MS analysis was performed on an Orbitrap Exploris 480 mass spectrometer (Thermo Fisher Scientific) equipped with an Easy n-LC 1200 high-performance liquid chromatography system (Thermo Fisher Scientific). The raw data from the Orbitrap Exploris 480 were analyzed with Proteome Discovery version 2.4.1.15 using the Sequest HT search engine for protein identification. The data from the in-gel digestion was then searched against the UniProt *Gallus gallus* protein database (updated September 2020). All nano LC-MS/MS experiments were performed by J. Wang (Laboratory of Proteomics, Institute of Biophysics, Chinese Academy of Sciences, Beijing 100101, China).

### Gel filtration chromatography

HEK293T cells were transfected with PA-Flag, PB1, PB2 of WSN virus, and avANP32A/B-V5. The intracellular polymerase complexes were purified using IP with Flag beads and subsequently loaded onto a Superose 6 Increase 10/300 GL (GE Healthcare). The fractions obtained were analyzed using Western blotting with anti-Flag and anti-V5 antibodies. The gel filtration calibration kit High Molecular Weight (HMW) (28403842, GE Healthcare) was used for marking the protein molecular weight.

### Passage of H9N2 viruses in MDCK cells

H9N2 viruses packaging avANP32A or huANP32A were obtained from HEK293T cells overexpressing avANP32A or huANP32A using the strict purification method described above. The titers of these two different viral stocks were then determined using standard methods in MDCK cells stably expressing avANP32A.

To perform passaging assays, confluent MDCK cells were inoculated for 2 hours with these two different H9N2 viral stocks at an MOI of 5. The cells were then washed and maintained with 1× DMEM [0.3% BSA and TPCK-treated trypsin (2 μg/ml)] for further 48 hours before collection of the supernatants for the next passage in MDCK cells. This procedure was repeated until the indicated passage.

### Animal experiments

Six-week-old female BALB/c mice were lightly anesthetized with CO_2_ and intranasally infected with a 10^6.4^ or 10^5.7^ EID_50_ of the indicated H9N2 virus. On day 2 and day 4 after infection, certain mice in each group were euthanized, and lung samples were collected for sequencing.

### Sequencing of viruses

RNA purification was carried out with the QIAamp Viral RNA Mini Kit (Qiagen) and reverse-transcribed into cDNA using a Uni12 primer (5′-AGCRAAAGCAGG-3′). For passage in MDCK, the C terminus of PB2 was amplified using the primers 798F (5′-CTCAATATGAGTGCAGACCGTGCT-3′) and 798R (5′-GATGACCATCCGAATCCTT-3′). Purification of the fragment using a Zymoclean Gel DNA recovery kit was followed by deep sequencing. An amplicon was formed by the ligation of Illumina adapter sequences and unique 8–base pair (bp) barcodes that differed by at least three bases. Pools of individuals were combined and run on an agarose gel, after which 798-bp fragments were manually excised and purified using a Zymoclean Gel DNA recovery kit. Each pool was amplified using 14 PCR cycles. DNA libraries were quantified using a high-sensitivity DNA analysis kit in a 2100 Bioanalyzer (Agilent Technologies). Pools were combined in equimolar concentration to form a single genomic library and were sequenced in one lane of a NovaSeq 6000 Illumina sequencer (paired-end, 2 × 150 bp). The reads were trimmed using AdapterRemoval-2.1.3 for adaptor sequences and for leading/trailing stretches of Ns. Subsequently, the remaining reads were filtered for quality and length using a window-based quality trimming algorithm. The window size was set to 5 bp and step size was set to 1 bp. Bases with a quality score of 2 or less were removed by trimming from the 3′ and only reads larger than 50 bp were kept. Illumina paired-end reads were aligned to the reference sequence using Burrows-Wheeler-Alignment Tool MEM (BWA-MEM) with the default parameters. Single-nucleotide polymorphisms and insertion/deletion (InDels) were called using Integrated Digital Error Supression (iDES) with subsequent filtering based on read map quality score, base quality score, and read depth. The sequences were processed and analyzed by Personalbio (Shanghai, China). Deep sequence data are deposited into the Sequence Read Archive with the accession code PRJNA1018500.

For the mouse experiment, DNA sequence spanning the PB2-627 and PB2-701 regions was amplified with specific primers using KOD Hot Start DNA polymerase. PCR products were purified using a DNA cleanup kit (Vazyme), followed by a T-A ligation for insertion into T vectors. After transformation, colonies were picked and sequenced.

### Quantification and statistical analysis

ImageJ software was used to measure the band intensity from the Western blot. GraphPad Prism was used for statistical tests. Statistical parameters are reported in the figures and figure legends. NS, not significant (*P* > 0.05), *P < 0.05, **P < 0.01, ***P < 0.001, and ****P < 0.0001.
