# Supplementary material for: Avian ANP32A incorporated in avian influenza A virions promotes interspecies transmission by priming early viral replication in mammals
Source: Sci Adv. 2024 Jan 31;10(5):eadj4163. doi: 10.1126/sciadv.adj4163 (PMC10830118; doi:10.1126/sciadv.adj4163)
Supplement: Supplementary file 1 — Figs. S1 to S6 Tables S1 to S3 [file sciadv.adj4163_sm.pdf]

Science Advances
AAAS

Supplementary Materials for

Avian ANP32A incorporated in avian influenza A virions promotes interspecies transmission by priming early viral replication in mammals

Lei Na *et al.*

Corresponding author: Xiaojun Wang, wangxiaojun@caas.cn

Sci. Adv. **10**, eadj4163 (2024)
DOI: 10.1126/sciadv.adj4163

This PDF file includes:

Figs. S1 to S6
Tables S1 to S3

Figure S1 Selection of an ANP32A polyclonal antibody that recognizing endogenous avANP32A

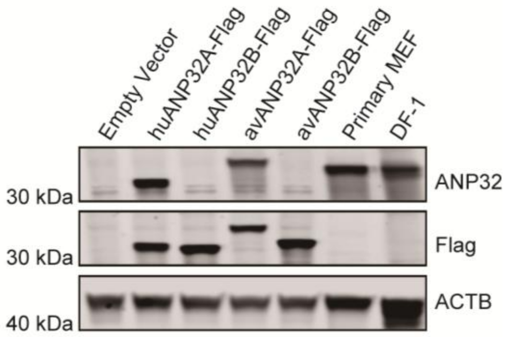

Western blot analysis of lysates from TKO cells transfected with the indicated Flag-tagged ANP32 plasmids, primary MEF cells and DF-1 cells.

Figure S2 Purification of IAV virions with a prior HAd to eliminate the contamination of exosomes.

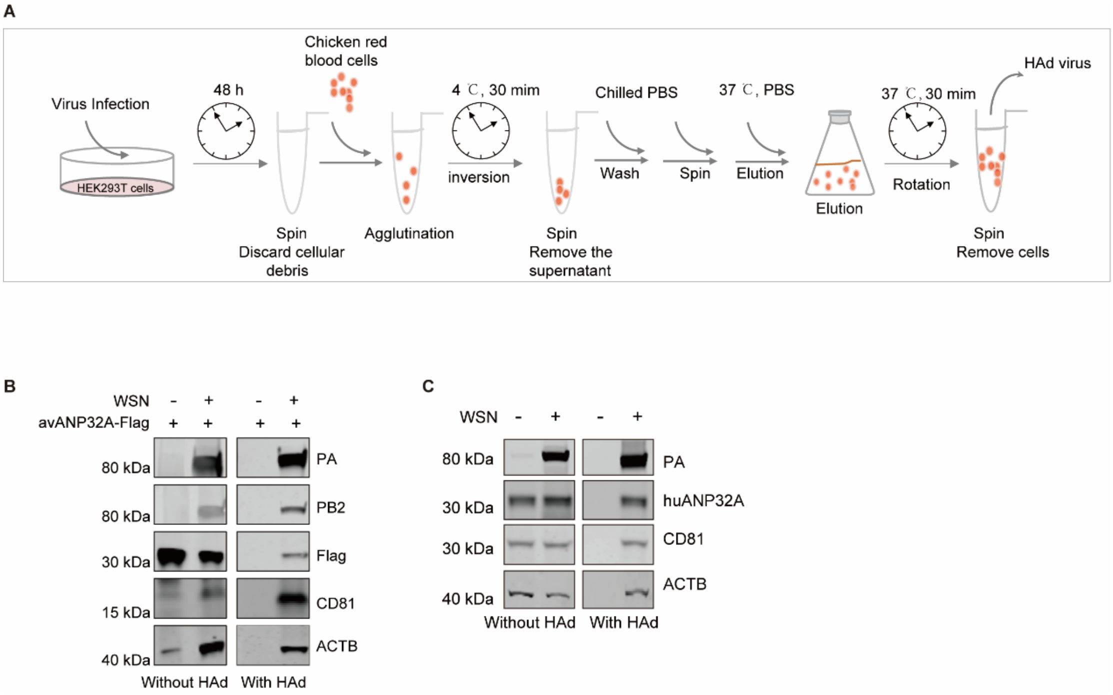

(**A**) Schematic diagram of the protocol for a haemadsorption/elution step (HAd). (**B)** HEK293T cells transfected with avANP32A-Flag constructs were infected with WSN virus (MOI = 0.01), and 48 h later, the supernatants from mock-infected and IAV-infected cells were pelleted by ultracentrifugation either with or without a prior HAd step. Afterwards, the pellets were resuspended and subjected to western blot analysis. (**C)** HEK293T cells were infected with WSN virus (MOI = 0.01), and 48 h later, the supernatants from mock-infected and IAV-infected cells were pelleted by ultracentrifugation either with or without a prior HAd step. Afterwards, the pellets were resuspended and subjected to western blot analysis.

Figure S3 Schematic representation of the protocol for the experiments shown in Fig. 4A

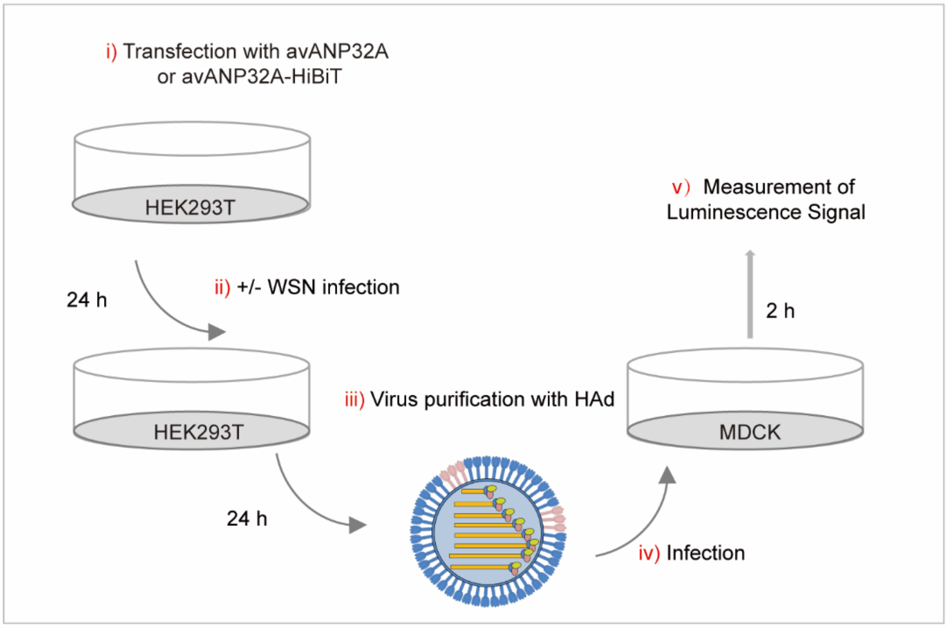

Figure S4 Establishment of an ANP32-dependent mini-genome assay in TKO cells

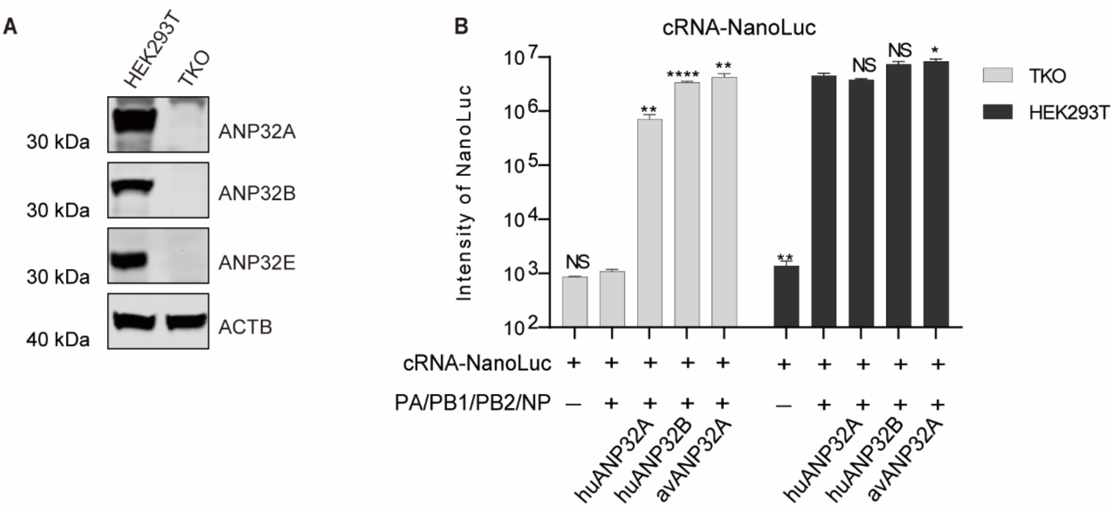

(A) Western blot analysis of ANP32A, ANP32B and ANP32E expression in TKO cells and their parental HEK293T cells. (**B)** IAV-cRNA-Nanoluc with or without plasmids encoding RNP proteins of WSN and indicated plasmids encoding different ANP32 proteins were transfected into HEK293T cells or TKO cells plated in 24-well plates. 24 h after transfection, cell supernatants were collected to measure the signal levels of NanoLuc. Significance was determined by unpaired Student's t-test against the group transfected with IAV-cRNA-NanoLuc together with RNP proteins of WSN within the same group (Error bars represent mean ± SEM from n = 3 independent biological replicates; NS, $p > 0.05$, *$p < 0.05$; **$p < 0.01$; ****$p < 0.0001$).

Figure S5 Avian ANP32 transferred by IBV supports influenza B virus replication in target cells

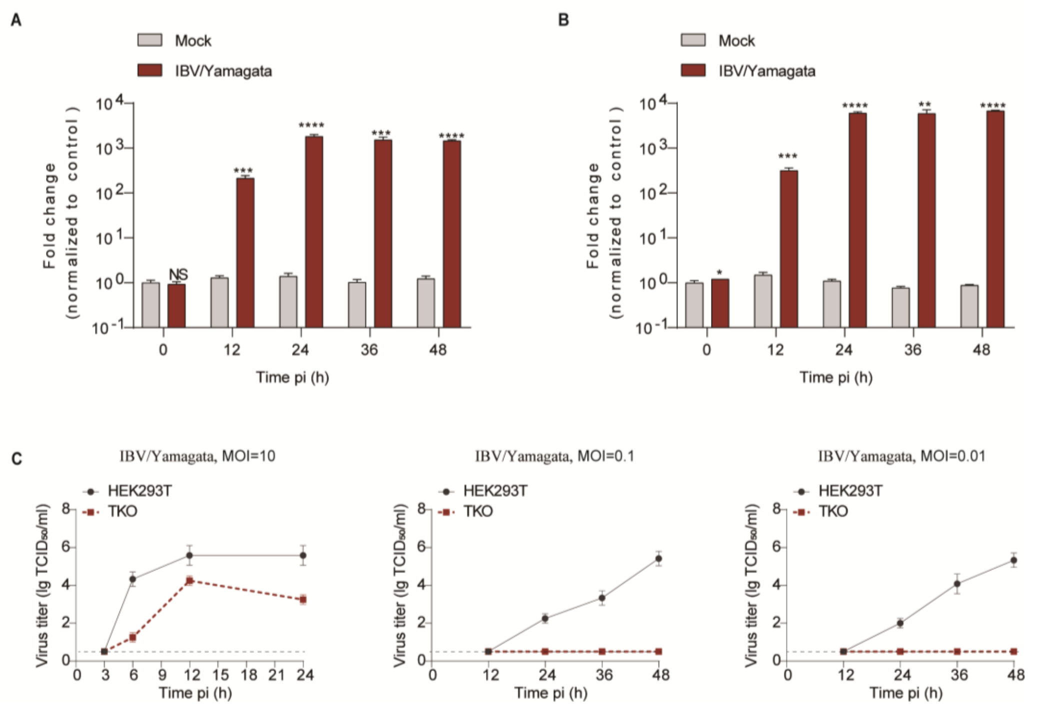

(A and **B)** Measurement of IBV/Yamagata polymerase activity in TKO cells transfected with vRNA-NanoLuc (A) and cRNA-NanoLuc (B) during IBV/Yamagata infection (MOI = 10) (Error bars represent mean ± SD from n = 3 independent biological replicates; unpaired t test; NS, $p > 0.05$, $^{**}p < 0.01$, $^{***}p < 0.001$, $^{****}p < 0.0001$). (**C**) Viral growth kinetics of IBV/Yamagata virus in HEK293T cells and TKO cells. The dashed line indicates the low limit of detection Date are presented as the mean ± SD (Error bars represent mean ± SD from n = 3 independent biological replicates).

Figure S6 Successful rescue WSN virus from TKO cells

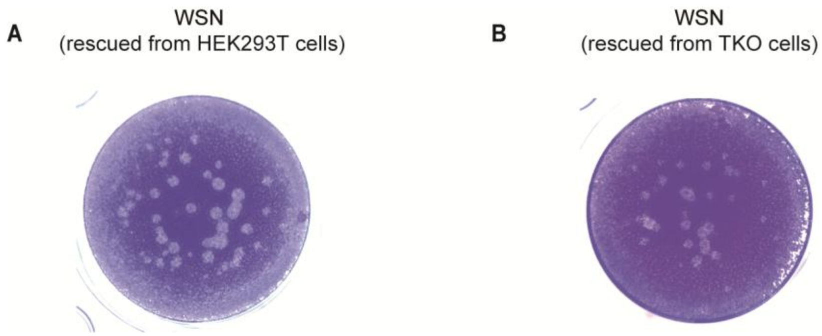

(**A** and **B**) Plaque phenotypes of WSN virus rescued from HEK293T cells (A) and TKO cells (B) separately.

Table S1 Assessment of the percentage of dots (ANP32 signal) within virions for Fig.1C

Rabbit IgG	Numeber of total Virions (N)		Number of virions with dots inside (M)	Ratio (total M/total N)
	Image 1	583	0	0 (0/2396)
	Image 2	491	0	
	Image 3	544	0	
	Image 4	478	0	
Rabbit ANP32A	Image 1	439	14	1.98% (46/2327)
	Image 2	346	20	
	Image 3	551	2	
	Image 4	502	5	
	Image 5	489	5	

Table S2 Assessment of the percentage of dots (ANP32 signal) within virions for Fig.1G

Mouse IgG	Numeber of total Virions (N)		Number of virions with dots inside (M)	Ratio (total M/total N)
	Image 1	42	0	0 (0/173)
	Image 2	68	0	
	Image 3	63	0	
Mouse Flag	Image 1	69	12	15.75% (95/603)
	Image 2	343	52	
	Image 3	191	31	

Table S3 Assessment of the presence or absence of the mutation in the eight gene segment of H9N2 virus recovered either from stock virus or samples at passage 6 and 9.

**Passage**	**Virus**	**Mutations in segment**							
		**NP**	**PA**	**PB1**	**PB2**	**NS**	**M**	**HA**	**NA**
**Stock**	H9N2(avANP32A)	None	None	None	None	None	None	None	None
	H9N2(huANP32A)	None	None	None	None	None	None	None	None
**P6**	H9N2(avANP32A)	None	None	None	1/4 627K<br>1/4 701N	None	None	None	None
	H9N2(huANP32A)	None	None	None	None	None	None	None	None
**P9**	H9N2(avANP32A)	ND	ND	ND	3/4 627K<br>1/4 701N	ND	ND	ND	ND
	H9N2(huANP32A)	ND	ND	ND	2/4 627K	ND	ND	ND	ND

Viral RNAs isolated from four populations of either H9N2 (avANP32A) or H9N2 (huANP32A) at passage 6 as well as viral RNAs isolated from the stock viruses for amplification of the eight gene segment. The PCR products were Sanger sequenced. For samples collected at passage 9, the Viral RNAs isolated from four populations of these viruses, and PCR products of the C-terminal of PB2 were Sanger sequenced as well. For values in parentheses, the number on the left of the slash shows the number of populations bearing indicated adaptative mutations, the number on the right of the slash shows the total number of populations of each virus. **ND**, not done; **None**, no mutation was observed.
